# Evaluation of a Nondestructive NMR and MRI Method for Monitoring the Drying Process of *Gastrodia elata* Blume

**DOI:** 10.3390/molecules24020236

**Published:** 2019-01-10

**Authors:** Yannan Chen, Hongjing Dong, Jingkun Li, Lanping Guo, Xiao Wang

**Affiliations:** 1 Key Laboratory of TCM Quality Control Technology, Shandong Analysis and Test Center, Qilu University of Technology (Shandong Academy of Sciences), Jinan 250014, China; 18954530681@163.com (Y.C.); donghongjing_2006@163.com (H.D.); ljk2091@163.com (J.L.); 2 College of Food Science and Engineering, Shandong Agricultural University, Tai’an 270018, China; 3 Resource Center of Chinese Materia Medica, State Key Laboratory Breeding Base of Dao-di Herbs, China Academy of Chinese Medical Sciences, Beijing 100700, China

**Keywords:** *Gastrodia elata* Blume, drying, LF-NMR, MRI, water variation, chemometric analysis

## Abstract

*Gastrodia elata* Blume (*G. elata*) is a prominent traditional herb and its dry tuber is officially listed in the Chinese Pharmacopoeia. To ensure the quality of dried *G. elata*, the establishment of a nondestructive and convenient method to monitor the drying process is necessary. In this study, a nondestructive low-field nuclear magnetic resonance (LF-NMR) and magnetic resonance imaging (MRI) method was introduced to monitor the drying process of *G. elata*. Three water states (bound, immobilized, and free) in *G. elata* samples were investigated through multiexponential fitting and inversion of the NMR data. The variation and distribution of the three water states during drying were monitored by LF-NMR, and the spatial distribution of water and internal structural changes were analyzed by MRI. Linear analysis of the moisture content, L* (lightness), b* (yellowness), and NMR parameters showed good correlations among them. Furthermore, partial least squares regression (PLSR) model analysis, which takes into account all NMR parameters, also showed good correlations among these parameters. All results showed that LF-NMR was feasible and convenient for monitoring moisture content. Therefore, LF-NMR and MRI could be used to monitor the moisture content nondestructively in the drying process of Chinese traditional herbs.

## 1. Introduction

Drying is an indispensable step in Chinese herbal medicine processing and is important for ensuring the quality [1]. Drying causes water evaporation to achieve the required moisture content and inhibits the biochemical reactions and proliferation of microorganisms [2]. Therefore, the drying degree (or moisture content) directly affects herb quality. Traditional methods for measuring the moisture content of Chinese herbal medicines include the weight method, differential scanning calorimetry, and near-infrared spectroscopy [3,4]. However, most of these methods are tedious, time-consuming, and interrupt the drying process. To reduce the drying time and obtain Chinese herbal medicines of good quality, it is necessary to develop a rapid and nondestructive method for analyzing water variations during the drying process.

Low-field nuclear magnetic resonance (LF-NMR) is a powerful tool for analyzing water variation in food owing to its nondestructive characteristics, rapid analysis speed, and low cost [5,6]. By measuring the proton relaxation times, the state, distribution, activity, and mobility of water can be determined using LF-NMR [7,8,9,10]. Magnetic resonance imaging (MRI), the other type of NMR technology, has been used to detect the internal distribution of water and structural changes during processing [11]. In recent years, combining NMR with MRI has been widely used to analyze food processing. Geng et al. [12] and Zhang et al. [13] have used both NMR and MRI to study dried sea cucumber rehydration and monitor the natto fermentation process, respectively. LF-NMR and MRI are also sensitive methods for monitoring water variation during drying processes. Cheng et al. [14] and Xu et al. [15] studied the water variation in shrimp and broccoli during drying, successfully applying these measurements to ensure food quality. However, there is no suitable method for analyzing the drying process of Chinese herbal medicines. Therefore, the development of a rapid and nondestructive analytical method for the Chinese herbal medicine industry would be extremely significant.

*Gastrodia elata* Blume (*G. elata*) is a prominent traditional herb used in many Asian countries [16]. The dry tuber of *G. elata* is officially listed in the Chinese Pharmacopoeia and has a beneficial effect on headaches, paralysis, rheumatism, and other nervous disorders [17,18,19]. To process *G. elata*, it must first be steamed and then dried to obtain high-quality pieces [20]. Therefore, monitoring the drying process of *G. elata* is important to obtain high-quality products. In this study, a nondestructive NMR and MRI method was introduced to monitor the drying process of *G. elata* by investigating water variation and internal structural changes. The relationships between moisture content, NMR parameters, and color parameters were investigated through linear analysis. Partial least squares regression (PLSR) analysis, taking into account all NMR parameters, was also applied. This NMR and MRI method could also potentially be applied to other Chinese herbal medicines.

## 2. Results and discussion

### 2.1. Effects of Steaming on Water States and Distribution

**Figure 1:**
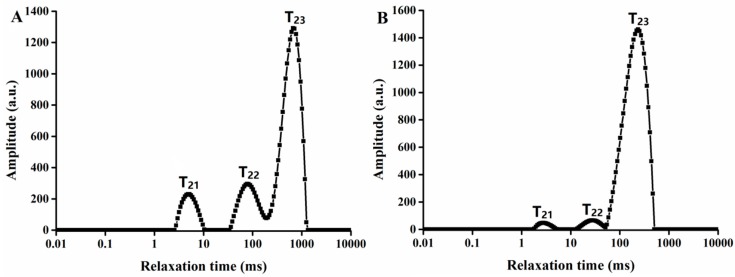
LF-NMR T_2_ relaxation time distribution curves of *G. elata* (**A**) before steaming and (**B**) after steaming.

Steaming *G. elata* is beneficial for drying [21]. Figure 1 shows the transverse relaxation time (T_2_) curves of fresh *G. elata* and *G. elata* after steaming. T_2_ reflects the chemical environment of protons and degree of water freedom in the sample [22]. The *G. elata* relaxation time curves showed three peaks, namely, T_21_ (0.1–10 ms), T_22_ (10–200 ms), and T_23_ (200–1000 ms) from left to right. These peaks represent the three molecular environments in which water molecules existed, where T_21_ corresponds to water binding closely to the macromolecular particles, T_22_ corresponds to water binding in the cytoplasm, and T_23_ corresponds to free water in the vacuole and intercellular space with strong mobility [23]. The corresponding signal amplitudes were A_21_, A_22_, and A_23_, respectively, representing the relative moisture contents of the three states.

Table 1 shows changes in the relaxation time and peak area of *G. elata* before and after steaming. After steaming, A_21_, A_22_, and A_23_ had all changed significantly, and the free water content had increased from 70.88% to 95.45%. Meanwhile, the bound water content had decreased from 11.11% to 1.62% and the content of immobilized water had decreased from 18.01% to 2.93%. These results indicated that the structure of *G. elata* was damaged by steaming, with water diffusing to the outside owing to differences between the internal and external pressures, while the free water content clearly increased.

**Figure 2:**
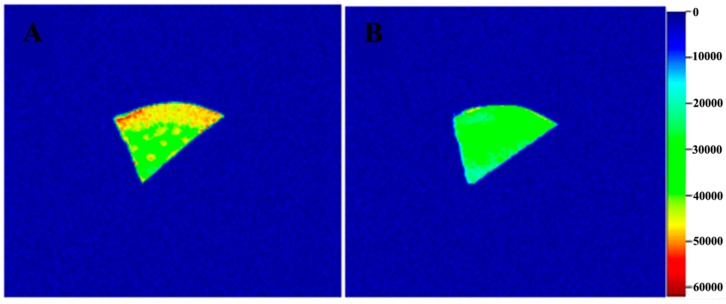
Cross-sectional MRI images of *G. elata* (**A**) before steaming and (**B**) after steaming.

Figure 2 shows cross-sectional MRI images of *G. elata* before and after steaming, where different colors represent different water contents [24]. From the color strip, red represents the highest moisture content and blue represents the lowest moisture content [25]. The water distribution inside *G. elata* was uneven before steaming, with the moisture content increasing gradually from inside to outside. The tissue structure was damaged after steaming, which accelerated the space movement of internal moisture, and the moisture distribution became uniform. These results demonstrated that steaming accelerated the drying rate.

### 2.2. Transverse Relaxation Time Analysis of G. elata after Steaming at Different Drying Temperatures

**Figure 3:**
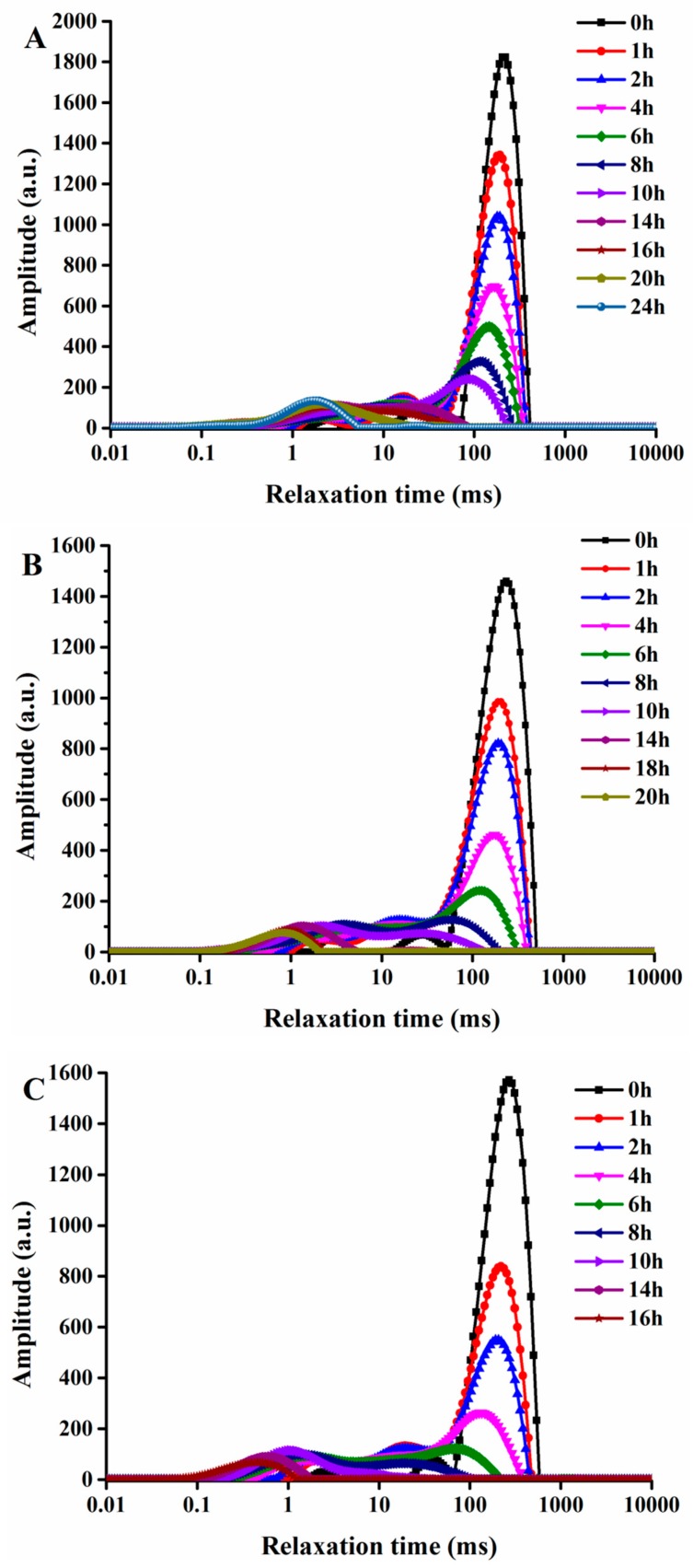
Transverse relaxation time curves of *G. elata* after steaming at different drying temperatures of (**A**) 40 °C, (**B**) 60 °C, and (**C**) 80 °C.

Figure 3 shows the transverse relaxation time curves of *G. elata* after steaming at different drying temperatures. The drying process changes the water content and states, and can also alter the water binding forces with macromolecules [26]. The peak area gradually decreased and the position of each peak gradually moved to the left. Meanwhile, the degree of water binding with non-water components became increasingly close, and the relaxation time gradually decreased.

Changes in the relaxation time and peak area of *G. elata* (after steaming) dried at 40, 60, and 80 °C are shown in Table 2, Table 3 and Table 4, which explain the changes in water states on the microscale. During the drying process, T_21_ values showed little change in the ranges 0.53–5.03 ms at 40 °C, 0.69–4.69 ms at 60 °C, and 0.93–4.20 ms at 80 °C. This indicated that bound water was tightly combined with the macromolecular substances and not easily removed during drying. The T_22_ and T_23_ values showed a significant decreasing trend with drying, which might be due to destruction of the cell membrane during drying. Macromolecules, such as polysaccharides, in *G. elata* flow into the intercellular space and increase the solution concentration, which increases opportunities for the combination of macromolecules with water that has poor mobility [27].

The signal amplitude of the T_2_ proton relaxation time was standardized against the sample mass, giving A_21_/g, A_22_/g, A_23_/g, and A_total_/g, which represent the signal amplitude per mass of bound water, immobilized water, free water, and total water, respectively [28].

As the area under the signal peak was proportional to the water content, it can be used to estimate the sample water content. The A_total_/g values continued to decline with increasing drying time, which indicated that water always moved in the direction of poor mobility. Furthermore, higher temperatures resulted in faster movement. The A_23_/g values decreased similarly to the A_total_/g values, which indicated that free water had the highest mobility and was removed first in the drying process. Changes in the A_22_/g values showed no obvious pattern, possibly owing to cell membrane damage caused by drying and the irregular movement of immobilized water. The A_21_/g values initially increased and then decreased in the drying process, which might be due to free water and immobilized water moving in the direction of poor mobility with increasing drying time, thus increasing the bound water content. After drying for 16 h, the contents of all three water states decreased, and only bound water remained at the end of drying.

### 2.3. MRI Analysis of G. Elata during Drying

**Figure 4:**
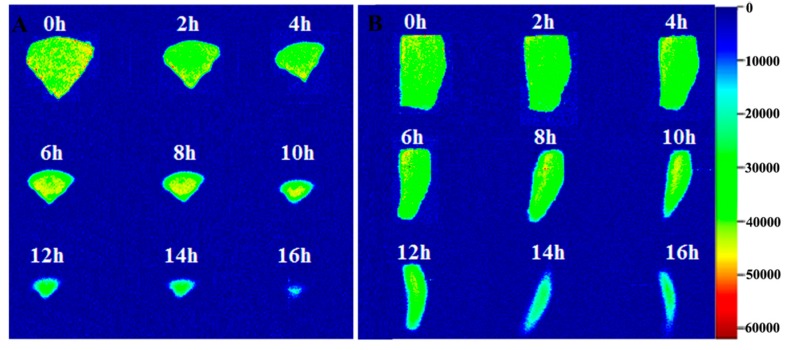
Vertical-sectional (**A**) and cross-sectional (**B**) MRI images of *G. elata* during the drying process at 60 °C.

As a fast and nondestructive analytical technology, MRI can analyze the internal structure and water states of samples during the drying process. Figure 4 shows vertical-sectional and cross-sectional MRI images of *G. elata* during the drying process. The color strip shows that different colors represent different proton densities and indirectly reflect moisture content. As shown in Figure 4, the color of the image was consistent after steaming *G. elata*, indicating that the water distribution was uniform. As the drying time increased, the yellow area decreased and the green area increased, indicating that moisture was continuously lost during the drying process. The proton density decreased from the outside to the inside, as previously observed in dried eggplant [29]. After drying for 16 h, the MRI image became barely visible, demonstrating that most of the water had evaporated, leaving only bound water that was tightly linked to the macromolecules.

### 2.4. Change in the Color of G. elata at Different Drying Temperatures

**Figure 5:**
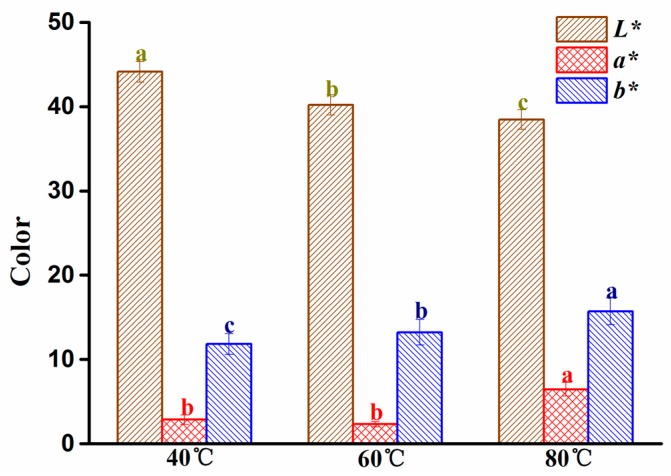
Effect of temperature on color parameters L*, a*, and b*. Different letters in the same color column indicate significant difference at *p* < 0.05.

The color of dry *G. elata* is another important indicator for evaluating herb quality. The impact of temperature on the color parameters (L*, a*, b*) is shown in Figure 5 L* values (lightness) decreased as the drying temperature increased (40, 60, and 80 °C), while b* values (yellowness) increased gradually, and a* values (redness) were significantly higher at 80 °C. These results might be attributed to the occurrence of Maillard browning reactions [30]. Therefore, it is advisable to use lower temperatures during the drying processing to ensure the quality of dried *G. elata* appearance is maintained. 

### 2.5. Correlation Analysis

As a fast and nondestructive analytical technique, NMR technology can replace some time-consuming methods if the relationships between NMR parameters and relative physicochemical parameters are determined. Relationships among moisture content (MC), LF-NMR parameters, and color parameters were investigated using linear analysis, with the corresponding correlation coefficients shown in Table 5. MC showed very significant correlations with T_21_, T_22_, A_23_/g, and A_Total_/g, with coefficients of 0.982, 0.980, 0.990, and 0.996, respectively. Similarly, L* showed very significant correlations with A_23_/g and A_total_/g, while b* showed significant correlations with T_22_ and T_23_. No significant correlation was found between a* and the NMR parameters in the *G. elata* samples. These results showed that LF-NMR was not only able to measure the moisture content, but also reflected the color of the medicinal material, which can reflect its overall quality.

### 2.6. Establishment of the PLSR Model

A strong correlation was found between A_total_/g and MC. Therefore, the linear equations can predict the moisture content in *G. elata.* However, as reported by Li et al. [31], the NMR parameters of T_2_ curves were extremely collinear. Therefore, it is necessary to establish a more reliable prediction method.

**Figure 6:**
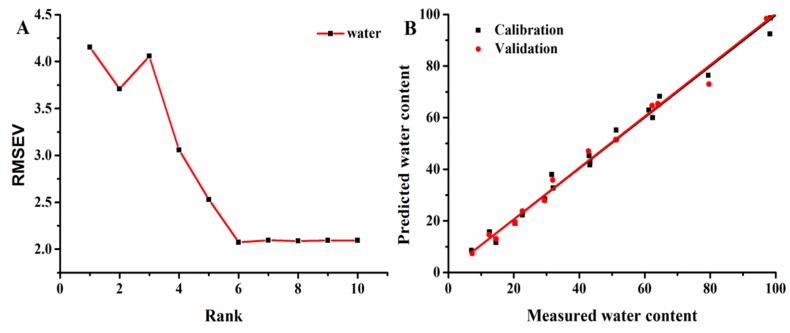
RMSEV of calibration vs. (**A**) rank values and (**B**) the measured and predicted plots of the PLSR models for water in *G. elata* samples.

As a reliable and convenient multivariate regression method, PLSR is well-suited to solving multicollinearity problems [32]. Figure 6A shows the relationship between the RMSEV of calibration and rank values, which clearly showed that the best rank value was 6. From the best rank value, predicted values vs. measured values of MC were obtained using the PLSR model. R^2^_c_, R^2^_cv_, RMSEC, RMSEV, and RPD were used to evaluate the model. The PLSR model for MC gave R^2^_c_ and R^2^_cv_ values of 0.994 and 0.993, with RMSEC and RMSEV values of 2.07 and 2.16, while the RPD value was 12.7. These results indicated that PLSR analysis, which accounted for all NMR parameters, also showed good correlations among these parameters. All results demonstrated that LF-NMR was a feasible and convenient method for monitoring moisture content.

## 3. Experimental

### 3.1. Sample Preparation

Fresh *G. elata* (second class *Gastrodia elata* f. glauca) used in this study was purchased from the Xiaocaoba Gastrodia Planting Base in Shaotong City (Shaotong, Yunnan Province, China) and stored in a refrigerator at 4 °C before use. Before each experiment, *G. elata* was washed and steamed for 10 min in a steamer (Qiaojieer Electric Co. Ltd., Zibo, Shandong, China). After steaming, the *G. elata* tuber was cut into pieces approximately 4 cm in height and 1 cm in radius with 90° curvature. The weight of each *G. elata* tuber sample was approximately 10 ± 0.5 g.

### 3.2. Moisture Content Measurement

The initial moisture content (MC) was calculated using Equation (1):

(1) $$\mathrm{MC}\;=\;\frac{W_{0}-W}{W}$$

where *W_0_* (g) is the initial mass and *W* (g) is the constant mass after drying at 105 °C.

### 3.3. Drying Process

*G. elata* tuber samples were laid flat on a metal grid tray and placed in an infrared-forced circulation drying oven (Wujiang Huafei Electric Heating Equipment Co. Ltd., Suzhou, China). The samples were dried at 40, 60, or 80 °C with a constant air velocity of 1 m/s over the tray. The samples were weighed every 30 min for the first hour, and then every 1 h until the change in weight between measurements was less than 0.01 g. All experiments were performed in triplicate.

### 3.4. LF-^1^H NMR Measurements

LF-^1^H NMR measurements were performed using an NMR Analyzer (MesoMR23-060H-I, Niumag Corp., Shanghai, China) equipped with a 0.5 T permanent magnet corresponding to a proton resonance frequency of 20 MHz at 32 °C. The *G. elata* tuber samples were placed in an NMR tube with an outer diameter of 25 mm. The proton decay signals were collected using the Carr–Purcell–Meiboom–Gill pulse sequence (CPMG) with a τ-value (time between 90° pulse and 180° pulse) of 200 μs, and 90° and 180° pulses of 7.5 and 15 μs, respectively. The NMR measurement parameters were set as follows: Echo time (TE), 0.35 ms; waiting time (TW), 2000 ms; data from 18,000 echoes acquired using 8 repeated scans.

Relaxation time analysis and distributed exponential curve fitting were performed using MultiExp Inv Analysis software (Niumag Corp., Shanghai, China). Multiexponential fitting analysis was performed on the relaxation data using a modified inversion algorithm to obtain improved fitting. The relaxation time and its corresponding water population (area ratio) from this analysis were recorded.

### 3.5. MRI Detection

MRI was performed using an NMR Analyzer (MesoMR23-060H-I, Niumag Corp., Shanghai, China) equipped with a 25-mm radio frequency coil at 32 °C. The MRI measurement parameters were set as follows: Slice width, 3 mm; slice gap, 1 mm; TR (time repetition), 2000; TE (time echo), 25 ms; average, 4.

### 3.6. Color Analysis

The fracture surface color of *G. elata* was measured using an NH310 high-quality portable colorimeter (Shenzhen 3NH Technology Co. Ltd., Shenzhen, China). The color was described in terms of L*, a*, and b*, where L* is lightness (0 for black, 100 for white), a* is green to red, and b* is blue to yellow. All experiments were performed in triplicate.

### 3.7. Statistical Analysis

Data are reported as means ± standard deviation. The moisture content, transverse relaxation time, and signal amplitude were calculated using the Statistical Package for the Social Sciences version 17.0 (SPSS Inc., Chicago, IL, USA). One-way analysis of variance (ANOVA) was used to determine whether differences between the mean values were significant. Origin 9 (OriginLab Corp., Northampton, MA, USA) was used to produce the figures. PLSR analysis was performed using Unscrambler 9.7 (Camo Software AS, Oslo, Norway). The performance of the PLSR model can be evaluated using the determination coefficients of calibration (*R^2^_C_*) and cross-validation (*R^2^_CV_*), and the root mean square errors of calibration (RMSEC) and cross-validation (RMSECV). In general, a good model should have high *R^2^_C_* and *R^2^_CV_* values, and low RMSEC and RMSECV values.

## 4. Conclusions

This study indicates that the LF-NMR and MRI method is feasible for monitoring water variation in *G. elata* samples during the drying process. Three water states (bound, immobilized, and free) in *G. elata* samples were investigated using multiexponential fitting and inversion of the NMR data. LF-NMR reflected the variation in water states in *G. elata* samples during drying, while MRI reflected the spatial distribution of water and structural changes. Significant correlations were found among MC, L*, b*, and NMR parameters. PLSR analysis, taking into account all NMR parameters, also showed good correlations among these parameters. These results suggested that LF-NMR was a feasible and convenient method for monitoring moisture content. This demonstrates that LF-NMR and MRI can monitor the drying process of *G. elata*, and has great potential for application to other Chinese herbal medicines.

## Figures and Tables

**Table 1:** Changes in relaxation time and peak area of *G. elata* before and after steaming.

	A_21_ (%)	A_22_ (%)	A_2 3_ (%)	T_21_ (ms)	T_22_ (ms)	T_23_ (ms)
Before steaming	11.11 ± 0.3^a^	18.01 ± 0.5^a^	70.88 ± 0.4^a^	5.18 ± 0.5^a^	96.81 ± 0.4^a^	584.94 ± 0.4^a^
After steaming	1.62 ± 0.2^b^	2.93 ± 0.6^b^	95.45 ± 0.5^b^	5.39 ± 0.3^a^	89.59 ± 0.5^b^	274.18 ± 0.6^b^

^a^ Values are means ± standard deviation (n = 3). ^b^ Different letters in the same column indicate a significant difference (*p* < 0.05).

**Table 2:** Changes in relaxation time and peak area of *G. elata* dried at 40 °C after steaming.

Drying Time (min)	T_21_ (ms) ^1,2^	T_22_ (ms) ^1,2^	T_23_ (ms) ^1,2^	A_21_/g ^1,2^	A_22_/g ^1,2^	A_23_/g ^1,2^	A_Total_/g ^1,2^
2	5.03 ± 0.15^a^	83.66 ± 4.66^a^	248.94 ± 3.65^a^	48.01 ± 1.91^f^	86.75 ± 3.22^e^	2210.86 ± 4.25^a^	2345.62 ± 6.23^a^
4	4.47 ± 0.19^b^	75.14 ± 5.31^b^	234.98 ± 4.60^b^	55.03 ± 3.56^f^	99.04 ± 2.45^d^	2015.86 ± 14.60^b^	2169.93 ± 4.26^b^
6	3.99 ± 0.21^c^	62.12 ± 4.57^c^	215.61 ± 3.73^c^	70.58 ± 4.04^e^	110.32 ± 4.08^c^	1878.33 ± 9.48^c^	2059.23 ± 3.58^c^
8	3.27 ± 0.20^d^	53.42 ± 2.61^d^	200.47 ± 4.26^d^	86.37 ± 4.41^d^	125.50 ± 3.97^b^	1650.33 ± 13.51^d^	1862.20 ± 3.22^d^
10	2.82 ± 0.14^e^	44.71 ± 3.07^e^	186.46 ± 2.99^e^	100.57 ± 4.08^c^	142.13 ± 2.78^a^	1433.37 ± 9.11^e^	1676.08 ± 8.21^e^
12	2.26 ± 0.26^f^	36.79 ± 2.86^f^	172.90 ± 6.29^f^	118.61 ± 6.25^b^	124.58 ± 4.20^b^	1116.68 ± 10.21^f^	1359.87 ± 6.58^f^
14	1.63 ± 0.17^g^	30.83 ± 1.33^fg^	155.35 ± 5.23^g^	135.29 ± 4.12^a^	95.40 ± 4.06^d^	882.68 ± 6.94^g^	1113.37 ± 7.66^g^
16	1.21 ± 0.22^h^	25.23 ± 1.64^gh^	136.09 ± 3.15^h^	108.95 ± 4.15^c^	104.71 ± 4.69^f^	544.84 ± 9.52^h^	758.50 ± 6.23^h^
18	0.96 ± 0.08^hi^	21.03 ± 1.55^hi^	121.28 ± 1.56^i^	73.95 ± 4.56^e^	56.61 ± 3.17^g^	212.35 ± 7.67^i^	342.91 ± 5.02^i^
20	0.76 ± 0.06^ij^	18.05 ± 1.36^i^	-	51.66 ± 2.63^f^	32.85 ± 1.86^h^	-	84.51 ± 2.18^j^
22	0.53 ± 0.08^j^	-	-	33.81 ± 3.33^g^	-	-	33.81 ± 1.47^k^

^1^ Values are means ± standard deviation (n = 3). ^2^ Different letters in the same column indicate a significant difference (*p* < 0.05).

**Table 3:** Changes in relaxation time and peak area of *G. elata* dried at 60 °C after steaming.

Drying Time (min)	T_21_ (ms) ^1,2^	T_22_ (ms) ^1,2^	T_23_ (ms) ^1,2^	A_21_/g ^1,2^	A_22_/g ^1,2^	A_23_/g ^1,2^	A_Total_/g ^1,2^
2	4.69 ± 0.35^a^	79.83 ± 5.47^a^	243.61 ± 4.48^a^	50.35 ± 4.09^f^	89.75 ± 4.03^d^	2129.84 ± 4.88^a^	2269.94 ± 12.43^a^
4	4.13 ± 0.60^ab^	70.13 ± 6.28^b^	226.65 ± 3.32^b^	58.03 ± 3.91^f^	102.37 ± 2.34^c^	1734.84 ± 8.75^b^	1895.25 ± 7.83^b^
6	3.69 ± 0.48^b^	59.77 ± 3.09^c^	208.94 ± 2.63^c^	75.253.40^e^	115.4 ± 4.17^b^	1417.91 ± 11.16^c^	1608.55 ± 6.25^c^
8	2.91 ± 0.16^c^	50.00 ± 4.05^d^	195.80 ± 6.31^d^	93.37 ± 4.21^d^	133.6 ± 5.11^a^	1088.21 ± 4.19^d^	1315.18 ± 7.56^d^
10	2.48 ± 0.34^cd^	41.83 ± 2.92^e^	186.46 ± 2.99^d^	105.25 ± 3.22^c^	114.71 ± 3.42^b^	829.41 ± 4.81^e^	1049.37 ± 5.28^e^
12	1.95 ± 0.13^d^	33.57 ± 1.51^f^	163.23 ± 7.05^e^	127.61 ± 5.27^a^	89.74 ± 2.40^d^	635.05 ± 8.62^f^	852.4 ± 4.33^f^
14	1.23 ± 0.28^e^	27.92 ± 0.97^fg^	145.35 ± 6.31^f^	115.29 ± 4.12^b^	92.35 ± 2.11^e^	415.83 ± 4.36^g^	623.48 ± 5.25^g^
16	1.07 ± 0.18^e^	22.73 ± 1.38^gh^	126.09 ± 3.15^g^	96.06 ± 5.44^d^	50.98 ± 1.17^f^	291.71 ± 6.90^h^	438.74 ± 3.28^h^
18	0.86 ± 0.12^e^	18.58 ± 2.15^h^	-	70.61 ± 5.37^e^	32.73 ± 4.25^g^	-	103.35 ± 3.22^i^
20	0.69 ± 0.05^e^	-	-	40.99 ± 3.30^g^	-	-	40.99 ± 2.13^j^

^1^ Values are means ± standard deviation (n = 3). ^2^ Different letters in the same column indicate a significant difference (*p* < 0.05).

**Table 4:** Changes in relaxation time and peak area of *G. elata* dried at 80 °C after steaming.

Drying Time (min)	T_21_ (ms) ^1,2^	T_22_ (ms) ^1,2^	T_23_ (ms) ^1,2^	A_21_/g ^1,2^	A_22_/g ^1,2^	A_23_/g ^1,2^	A_Total_/g ^1,2^
2	4.20 ± 0.14^a^	73.84 ± 4.06^a^	226.27 ± 5.32^a^	57.68 ± 1.93^d^	91.08 ± 4.04^c^	2026.95 ± 6.34^a^	2175.71 ± 9.82^a^
4	3.70 ± 0.49^a^	65.46 ± 3.67^b^	209.65 ± 4.18^b^	72.70 ± 4.75^c^	114.61 ± 3.13^b^	1546.24 ± 10.91^b^	1733.55 ± 8.43^b^
6	2.97 ± 0.22^b^	55.10 ± 3.72^c^	198.94 ± 5.81^b^	90.58 ± 7.13^b^	125.40 ± 3.99^a^	1136.94 ± 11.04^c^	1352.92 ± 7.26^c^
8	2.47 ± 0.24^bc^	46.34 ± 4.36^d^	185.32 ± 4.90^c^	113.37 ± 5.79^a^	116.93 ± 4.75^b^	774.60 ± 6.61^d^	1004.9 ± 5.25^d^
10	1.98 ± 0.18^cd^	37.17 ± 1.93^e^	166.81 ± 3.20^d^	96.92 ± 4.59^b^	88.04 ± 2.04^c^	570.56 ± 5.58^e^	755.52 ± 6.47^e^
12	1.64 ± 0.24^d^	28.57 ± 1.38^f^	143.23 ± 7.05^e^	79.28 ± 2.89^c^	63.07 ± 2.58^d^	269.21 ± 4.21^f^	411.56 ± 5.23^f^
14	1.03 ± 0.15^e^	21.58 ± 3.70^f^	-	55.96 ± 4.15^d^	35.67 ± 3.69^e^	-	91.62 ± 4.22^g^
16	0.93 ± 0.22^e^	-	-	35.39 ± 3.51^e^	-	-	35.39 ± 3.21^h^

^1^ Values are means ± standard deviation (n = 3). ^2^ Different letters in the same column indicate a significant difference (*p* < 0.05).

**Table 5:** Correlation coefficients among moisture content (MC), LF-NMR parameters, and color parameters.

	T_21_	T_22_	T_23_	A_21_/g	A_22_/g	A_23_/g	A _Total_/g
MC	0.982 *	0.980 **	0.972 *	0.233	0.131	0.991 **	0.996 **
L*	−0.162	−0.628	−0.729	0.527	0.521	0.904 **	0.913 **
a*	0.017	−0.219	0.228	0.509	0.432	0.613	0.722
b*	0.728	0.823 *	0.845 *	0.749	0.071	0.769	0.776

* *p* < 0.05, significant at the 0.05 level, ** *p* < 0.01, significant at the 0.01 level.
